# Mediterranean Dietary Pattern and Psychosocial Health Problems in Spanish Adolescents: The EHDLA Study

**DOI:** 10.3390/nu15132905

**Published:** 2023-06-27

**Authors:** Sofía Alfaro-González, Miriam Garrido-Miguel, Vicente Martínez-Vizcaíno, José Francisco López-Gil

**Affiliations:** 1 Health and Social Research Center, Universidad de Castilla La-Mancha, 16071 Cuenca, Spain; sofia.alfaro@alu.uclm.es (S.A.-G.); vicente.martinez@uclm.es (V.M.-V.); 2 Facultad de Enfermería, Universidad de Castilla-La Mancha, 02006 Albacete, Spain; 3 Facultad de Ciencias de la Salud, Universidad Autónoma de Chile, Talca 3460000, Chile; 4 Navarrabiomed, Hospital Universitario de Navarra (HUN), Universidad Pública de Navarra (UPNA), IdiSNA, 31006 Pamplona, Spain; josefrancisco.lopez@uclm.es; 5 Department of Environmental Health, Harvard University T.H. Chan School of Public Health, Boston, MA 02138, USA; 6 One Health Research Group, Universidad de Las Américas, Quito 170124, Ecuador

**Keywords:** adolescence, eating healthy, KIDMED, Mediterranean diet, mental health, youths

## Abstract

The aims of the present study were twofold: to determine the association between adherence to the Mediterranean diet (MedDiet) and psychological problems and to assess the relationship between different food groups of the MedDiet and different patterns of psychosocial health problems in a sample of Spanish adolescents from the Valle de Ricote (Region of Murcia, Spain). This cross-sectional study included a representative sample of 700 adolescents (57% girls) between 12 and 17 years of age. Adherence to the MedDiet was assessed by the Mediterranean Diet Quality Index for Children and Teenagers (KIDMED). Psychosocial health problems were measured by the 25-item self-report version of the Strengths and Difficulties Questionnaire (SDQ). ANCOVA models were used to test the mean differences in psychosocial health problems (SDQ subscales) by adherence to MedDiet categories (low, medium, and high). Multiple linear regression was used to determine the association between different food groups of the MedDiet and psychosocial health problems. Adolescents with low adherence to the MedDiet had significantly higher scores in SDQ total difficulties, conduct problems, and hyperactivity problems and lower scores in pro-social behavior (*p* < 0.05) than their peers with medium and high adherence to the MedDiet. In relation to specific MedDiet food groups, fruit, nut, and legume intake were associated with higher psychosocial health. However, sweets, skipping breakfast, or high consumption of pasta or rice (≥5 weeks) were associated to higher psychosocial health problems. Our results show that adolescents with higher adherence to the MedDiet could benefit from greater psychosocial health. Individually, some patterns of MedDiet, such as fruit, nut, and legume intake, may reduce psychosocial health problems. These cross-sectional results should be confirmed in longitudinal and intervention studies.

## 1. Introduction

Psychosocial health is broadly defined as including both social and psychological outcomes. However, there is no consensus on this definition in this field [1]. It is also important to highlight that the presence of psychosocial health problems, such as conductual or emotional problems during adolescence, is closely related to the development of major mental health problems later in life [2]. Behavior and emotional disorders (including anxiety–depressive disorders) affect 10–20% of children and adolescents worldwide and account for a large portion of the global burden of disease [3]. Adolescence is the transitional stage of development between childhood and adulthood and is characterized by relevant physical and psychological changes [4]. In this sense, adolescence is a critical period, since half of all lifelong mental health problems begin before the age of 18 years [5]. Thus, the prevention of psychosocial health problems during adolescence through the identification of potentially related modifiable factors is crucial to improving long-term mental health.

The etiology of mental disorders includes genetic, biological [6], environmental, low socioeconomic level [7], disaster events such as the COVID-19 pandemic [8,9,10,11], and lifestyle factors, such as poor physical activity [12] and nutritional quality diet [13]. In relation to diet, several studies have shown that Western and unhealthy diets are associated with an increase in mental health problems, including hyperactivity disorders and conductive problems in adolescents [13,14,15,16,17,18,19,20]. Different pathways have been proposed, suggesting that inflammatory processes are associated to unhealthy and processed food consumption and could describe part of the relationship between unhealthy diet and mental health [21,22]. Otherwise, numerous studies have also shown that the consumption of certain types of foods that are part of healthy dietary patterns (such as fruits, whole grains, vegetables, nuts, fish, and olive oils, among others) could notably improve mental health, in part because of their high content of certain nutrients, such as folate, magnesium, b-group vitamins, selenium, zinc, mono- and polyunsaturated fatty acids, polyphenols, and fiber [23,24,25].

Current evidence reports the positive health impacts of the Mediterranean diet (MedDiet), such as a decline in the risk of cardiovascular disease [26], various types of cancer [27], academic performance [28], depression, type 2 diabetes mellitus [29], and mental disorders [29]. The MedDiet represents a traditional healthy dietary pattern characterized by high consumption of fruit, vegetables, cereals with whole grains, nuts, legumes, fish, and unsaturated fat such as olive oil. It also includes moderate consumption of dairy foods and low consumption of processed and red meat products, saturated lipids, and sweets [29]. Due to its excellent composition of foods and nutrients, adherence to the MedDiet could also have protective effects on mental health. On this point, the latest studies have reported an inverse relationship between adherence to the MedDiet and depressive and anxiety disorders, but such findings have shown contradictory results [30,31,32]. Therefore, further studies are required to explain and reinforce the evidence about the relationship between the MedDiet and different mental health problems. Additionally, most studies have focused on studying the main mental disorders (e.g., depression and anxiety) in the adult population. Thus, further studies are required to reinforce the evidence between adherence to the MedDiet and different psychosocial health problems, specifically in the adolescent population group. Therefore, the objectives of this cross-sectional study were twofold: (i) to analyze the relationship between adherence to the MedDiet and psychological problems and (ii) to assess the association between different food groups of the MedDiet and different patterns of psychosocial health problems in a sample of Spanish adolescents.

## 2. Materials and Methods

### 2.1. Study Design and Population

This is a secondary analysis using data from the Eating Healthy and Daily Life Activities (EHDLA), which involve a representative sample of adolescents (aged 12–17 years) from the Valle de Ricote (Region of Murcia, Spain). The following secondary schools were evaluated for this study: CE El Ope, IES Vicente Medina, and IES Pedro Guillén. All data collection took place during the 2021/2022 academic year. The described methodology of this project, including the calculation of the sample size [33], has been published elsewhere [34]. For this study, a total sample of 700 adolescents (57% girls) was examined.

Considering contribution in this study, the parents or legal guardians of the adolescents signed a written informed consent form. Participants were also informed about the project and were asked about their disposition to join in the study.

The inclusion criteria were the following: (1) adolescents between 12 and 17 years, who (2) lived and/or were registered in Valle de Ricote. In referring to exclusion criteria, students were not included in the study when they (1) were excused from the subject of physical education at secondary school, since both the tests and the fulfilment of the questionnaires were performed during the physical education lessons, (2) had any pathology that required special consideration, (3) were under pharmacological treatment, (4) did not consent to participate in the project, or (5) had parents or legal guardians who did not permit them to take part in the study.

Ethics approvals were conducted according to the Ethics Committee of the University of Murcia (ID 2218/2018) and the Ethics Committee of the Albacete University Hospital Complex and the Albacete Integrated Care Management (ID 2021-85). It will be executed following the Helsinki Declaration, regarding the human rights of the participants included in the study.

### 2.2. Study Variables

#### 2.2.1. Psychosocial Health Problems (Dependent Variable)

The 25-item self-report version of the Strengths and Difficulties Questionnaire (SDQ) was assessed for psychosocial health problems for children and adolescent mental health problems between 4 and 17 years old [35]. It is applied for clinical evaluation, screening psychiatric disorders, and epidemiological study. The SDQ incorporates five scales: (i) emotional symptoms, (ii) conduct problems, (iii) hyperactivity, (iv) peer problems, and (v) pro-social behavior (reverse scored). The 25 items were scored from 0 to 2 points responded with a 3-point scale, “certainly true”, “somewhat true”, and “not true”. In addition, the cutoff data scores were categorized by no psychosocial health problems (normal and borderline) and psychosocial health problems (abnormal).

#### 2.2.2. Adherence to the Mediterranean Diet (Independent Variable)

Mediterranean Diet Quality Index for Children and Teenagers (KIDMED) was used to assess adherence to the MedDiet [36]. The KIDMED index was earlier approved and commonly used in the young Spanish population [28,37,38,39,40]. The KIDMED index is based on a 16-question test ranging from 0 to 12. Items describing unhealthy characteristics related to the MedDiet are scored with –1 point, and those describing healthy characteristics are scored with +1 point. Three categories’ levels were categorized by the total of all scores: (a) low, low diet quality MedDiet (≤3 points); (b) moderate, needed to increase MedDiet (4–7 points); and (c) high, optimal MedDiet (≥8 points).

#### 2.2.3. Covariates

Birth date and sex were reported by themselves. Age was assessed from the birth date. The body weight of adolescents was measured with an electronic scale (with an exactitude of 0.1 kg) (Tanita BC-545, Tokyo, Japan), while a portable height rod was used for height (with an exactitude of 0.1 cm) (Leicester Tanita HR 001, Tokyo, Japan). Body mass index (BMI) was evaluated by dividing body weight (kg) by height (m^2^). The family affluence scale (FAS-III) was used for socioeconomic status (SES) [41]. 

Sedentary behaviors including physical activity were evaluated by the Youth Activity Profile Physical (YAP) questionnaire [42]. The YAP is a self-administered, 7-day (previous week) recall questionnaire for children and adolescents (aged 8–17 years) composed of 15 items. The items use a five-point Likert scale and are divided into three categories: (1) activity school (transportation to and from school, lunch, and activity during physical education and recess); (2) activity out of school (activity before school, activity right after school, activity during the evening, and activity on each weekend day); and (3) sedentary habits (sedentary habits related to watching television, playing videogames, or using smartphones) [41]. Physical activity (at school and out of school) and sedentary behavior (sedentary habits) scores were determined by summing the items in each section. The Spanish version of YAP (YAP-S) was validated and adapted before [43].

Sleep duration was evaluated by asking, “What time do you usually go to bed?” and “What time do you usually get up?” The following formula, ((average nocturnal sleep duration on weekdays × 5) + (average nocturnal sleep duration on weekends × 2))/7, was used to determine the average daily sleep duration for each adolescent.

Tobacco status and alcohol consumption status were categorized as follows: no (never) or yes (from 1 to 30 days).

### 2.3. Statistical Analysis

Means (M) and standard deviation (SD) or frequencies (n) and percentages (%) are reported for all quantitative or qualitative data, respectively. The Kolmogorov‒Smirnov test (*p* > 0.05) had been used for verification of variables’ normality distribution. In addition, analyses of covariance (ANCOVA) were carried out to evaluate the mean differences between psychosocial health problems (SDQ subscales) (as dependent variables) by adherence to MedDiet categories (low, medium, and high). A post hoc Bonferroni test was used for post hoc pairwise comparisons. Furthermore, multiple regression analyses using the backward elimination method (*p* for removing a variable ≥ 0.10) were also used to determine the association between all food groups included in the KIDMED questionnaire and each psychosocial health problem domain of the SDQ individually (as dependent variables) among Spanish adolescents. Standardized *β* coefficients and *R*^2^ values are reported. Linear regression models were validated previously (linearity, independence, homoscedasticity, normality, and noncollinearity). Given that we did not release a relationship between adherence to the MedDiet and psychosocial health problems in relation to sex (*p* = 0.925), all the analyses were conducted together.

Age, sex, waist circumference, socioeconomic status, YAP-S physical activity, YAP-S sedentary behaviors, sleep duration, alcohol consumption, and tobacco consumption were adjusted for analyses. Statistical significance was evaluated at a *p*-value ≤ 0.05. SPSS software (IBM Corp., Armonk, NY, USA) for Windows (version 28.0) was used for all analyses.

## 3. Results

Characteristics of the adolescents in the study was described in Table 1. A total of 700 (399, 57%, girls) with a mean age of 14.0 (SD = 1.5) were analyzed. The KIDMED mean score was 6.5 (SD = 2.6). One the one hand, 37.3% of adolescents had high adherence to the MedDiet. On the other hand, 15.7% of adolescents showed emotional symptoms, 11.9% had conduct problems, 14.9% had hyperactivity problems, and 6.1% had peer problems.

**Figure 1:**
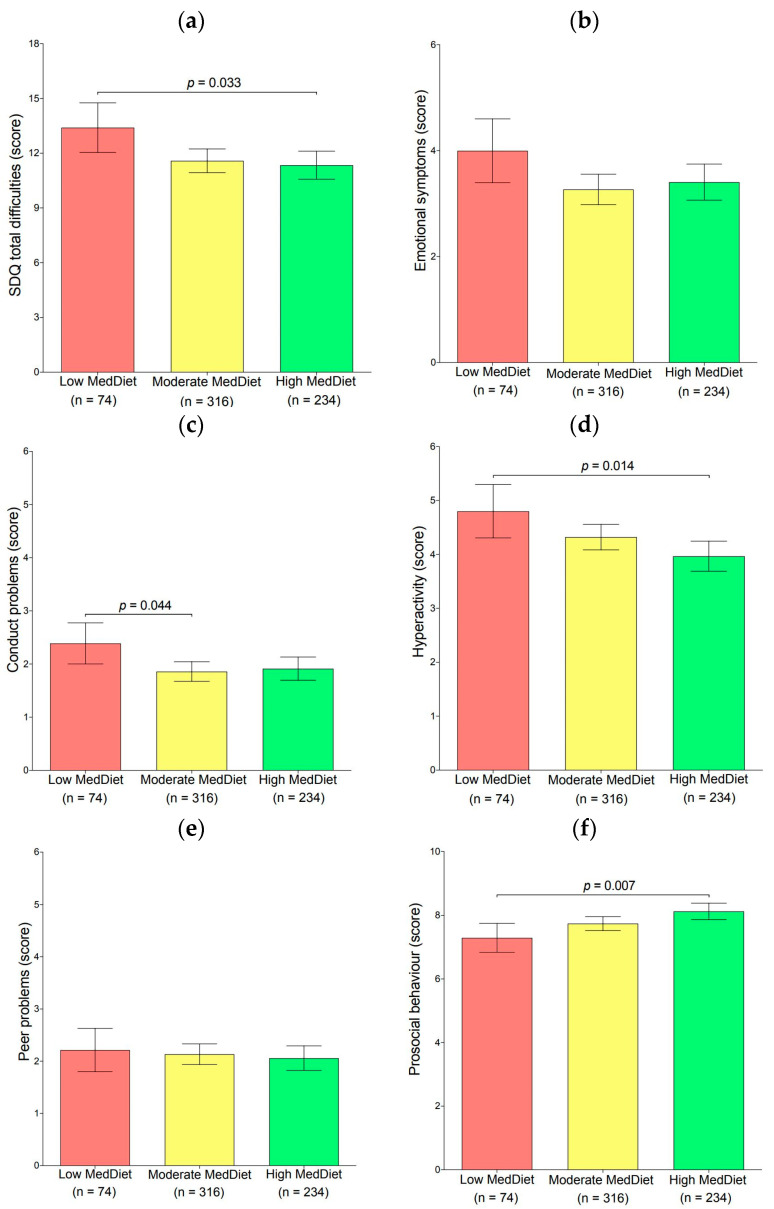
Mean differences between (**a**) the SDQ total difficulties, (**b**) emotional symptoms, (**c**) conduct problems, (**d**) hyperactivity problems, (**e**) peer problems, and (**f**) pro-social behavior by MedDiet categories. Analysis of covariance was adjusted for age, sex, waist circumference, socioeconomic status, YAP-S physical activity, YAP-S sedentary behaviors, sleep duration, total energy intake, alcohol, and tobacco consumption. Brackets designate significant differences in the means (*p* < 0.05) between categories by the Bonferroni multiple comparison post hoc test. Error bars represent the standard error.

ANCOVAs were performed to examine the mean differences in the individual domains of the SDQ questionnaire, as well as total difficulties of psychosocial health problems by adherence to the MedDiet categories (low, medium, and high). The outcomes of the adjusted models are represented in Figure 1. Adolescents who had low adherence to the MedDiet had significantly higher scores in SDQ total difficulties, conduct problems, and hyperactivity problems and lower scores in pro-social behavior (*p* < 0.05) than those who had high adherence to the MedDiet.

Table 2 describes the association between different food groups included in the KIDMED questionnaire and psychosocial health problems (SDQ subscales) among Spanish adolescents. The standardized *β* coefficients for which statistical associations were found were as follows: fruit, or fruit juice daily (conduct problems: *β* = −0.084, *p* = 0.032; SDQ total difficulties: *β* = −0.082, *p* = 0.039), second fruit daily (hyperactivity problems: *β* = −0.132, *p* = < 0.001), fresh or cooked vegetables >1/day (peer problems: *β* = 0.108, *p* = 0.007; SDQ total difficulties: *β* = 0.087, *p* = 0.026), pulses >1/day (prosocial scale: *β* = 0.153, *p* = < 0.001), pasta or rice ≥ 5/week (conduct problems: *β* = 0.092, *p* = 0.019), nuts regularly (emotional problems: *β* = −0.138, *p* = < 0.001; SDQ total difficulties: *β* = −0.099, *p* = 0.010), sweets and candy daily (hyperactivity problems: *β* = 0.105, *p* = 0.010), and no breakfast (emotional problems: *β* = 0.086, *p* = 0.029; conduct problems: *β* = 0.091, *p* = 0.024; SDQ total difficulties: *β* = 0.100, *p* = 0.013).

## 4. Discussion

Our findings highlight that Spanish adolescents with low adherence to the MedDiet had higher conduct problems, hyperactivity problems, and total difficulties in comparison with their counterparts with moderate and high adherence to the MedDiet. Conversely, adolescents with low adherence to the MedDiet showed lower scores in pro-social behavior. In relation to specific MedDiet patterns, fruit, nut, and legume intake were associated with higher psychosocial health specifically improved conduct problems, emotional problems, and prosocial behavior domains, respectively. However, sweets, skipping breakfast, or high consumption of pasta or rice (≥5 weeks) were related to higher psychosocial health problems, specifically in the domains of emotional problems, conduct problems, hyperactivity problems, and total difficulties. The findings of our cross-sectional relationship between healthy diet patterns and psychosocial health problems are concordant with other similar studies in adolescents from Australia [44] and England [13]. Jacka et al. [13] showed that adolescents who had a higher score in unhealthy diet were more than twice as expected to be symptomatic on the SDQ. However, the English study described weaker evidence between healthy diet score and behavioral problems.

Slightly more than half of our adolescents (50.4%) had moderate adherence to the MedDiet, and only 37.4% of adolescents had good adherence to the MedDiet. These results are similar to other studies in adolescents in Spain, as well as in the Region of Murcia [28,37,38,45,46]. However, these data had higher rates of adherence to the MedDiet than studies carried out in other countries [47,48,49,50]. The reduction in adherence to the MedDiet could be because of the high intake of unhealthy diets, such as the Western diet [47]. Western diet is characterized by ultra-processed food, high consumption of processed red meat, soft and energy drinks, refined grains, high intake of sweets, and low consumption of fruit, vegetables, and legumes [51]. The consumption of ultra-processed food could replace healthy food presented in the MedDiet [52]. Several studies have shown that consuming sweets and candy could be associated with behavioral problems, especially hyperactivity [53,54,55]. Additionally, in recent decades, adolescents have increasingly consumed energy drinks. These drinks could produce cardiovascular, mental, and sleep problems in adolescents [56]. A systematic review of observational studies has shown a positive association between consuming sugar and symptoms of attention-deficit/hyperactivity disorder (ADHD). Similar results were shown in our adolescents; the intake of sweets and candy several times was positively associated with hyperactivity disorder.

Different components of the MedDiet have been associated with better mental health. Fruits are one of the most important foods from the MedDiet and are characterized as a source of fiber, nutrients such as vitamin C or potassium, and polyphenols [57]. In our results, fruits were inversely associated with higher conductual problems, hyperactivity problems, and SDQ total difficulties. Findings from an Australian study reported that consuming fruit in children and adolescents had higher values in SDQ total difficulties [44]. Additionally, a systematic review showed that the consumption of fruit is protective from the risk of mental symptoms [58].

Other important elements of the MedDiet are nuts. It has multiple benefits for our system, such as cardiovascular and cancer prevention [59,60] or mental health [61]. It includes monounsaturated (MUFA) and polyunsaturated (PUFA) fatty acids such as α-linolenic acid (ALA), vegetable proteins, fiber, B-vitamin folate, vitamin E, minerals, and polyphenols, among others [59,60]. PUFAs and polyphenols are considered the best composition for better mental health. Studies have highlighted the importance of consuming nuts for better cognitive function, such as dementia outcomes or depression [61,62]. However, the evidence of the studies was poor. The consumption of nuts in our results is associated with lower emotional problems and SDQ total difficulties. The findings of different studies have also reported lower values of emotional symptoms with regular nut consumption [23,63].

On the other hand, consumption of whole-grain cereals has multiple benefits for health, such as better intestinal flora diversity, regulation of plasma glucose, and prevention of cardiovascular diseases or chronic metabolic diseases. These benefits are due to the consumption of dietary fiber, proteins, polyphenols, and β-glucans present in whole grain cereals [64]. Regarding mental health, a Japanese study of children reported that the consumption of cereals or rice in children reduces behavioral problems [65]. However, another cross-sectional study from the United Kingdom showed no relation between consuming cereals and the association of mental health symptoms [66]. Our results showed that a high consumption of pasta or rice (≥5 weeks) is associated with higher values in psychosocial health problems, specifically conduct problems. It is important to know that in our study, the type of cereal that adolescents consumed, whole cereal or refined cereal, is unknown. Refined cereals had lower fiber and micronutrient contents than whole cereal [67]. Additionally, refined cereals and sugar have similar metabolic compositions, producing high glycemic and plasma insulin levels [68]. More studies are needed to determine the association between the intake of pasta or rice and mental health.

There were some limitations in our study. This is a cross-sectional study that does not enable for a cause‒effect association between the MedDiet and psychosocial health problems. Further studies with different designs (e.g., experimental or longitudinal studies) are required to examine whether the MedDiet and its components reduce psychosocial health problems. However, clinical trials are required to establish a causal impact of the MedDiet on psychosocial health problems in adolescents; the available data illustrate a possible association between a healthy diet and psychosocial health problems [13,44]. The information on the KIDMED questionnaire was self-reported and is subject to recall and reporting biases by adolescents. However, the KIDMED questionnaire is an instrument validated in Spain. Even though our analyses controlled for sociodemographic, anthropometric, and lifestyle covariates (e.g., physical activity, sedentary behavior, and sleep duration), residual confounding is still possible. Conversely, one strength of the study is that we used validated measures to determine psychosocial health problems (i.e., SDQ questionnaire). The second strength is the association of MedDiet and psychosocial health problems in adolescents because adolescents are an understudied population. Nevertheless, these results should be cautiously interpreted because of potential bias such as the lack of representativeness of the sample that prevents generalizing the results.

## 5. Conclusions

Adolescents with greater adherence to the MedDiet could benefit from greater psychosocial health. Individually, some patterns of the MedDiet, such as fruit, nut, and legume intake, may reduce emotional, conduct, hyperactivity problems, and prosocial behavior. However, skipping breakfast, intake of sweets, and a high intake of rice and pasta could increase emotions, conduct, hyperactivity, and peer problems. Notwithstanding, these cross-sectional results should be confirmed in longitudinal and intervention studies. Our outcomes support the importance of consumption in public health methods to improve adolescents’ psychosocial health problems and prevent future mental disorders such as depression, stress, or anxiety.

## Figures and Tables

**Table 1:** Descriptive characteristics of the participants (N = 700).

Variables	M ± SD/*n* (%)
Sex	
Boys	301 (43.0)
Girls	399 (57.0)
Age (years)	14.0 ± 1.5
Recruiting school	
CE El Ope, n (%)	151 (21.6)
IES Vicente Medina, n (%)	355 (50.7)
IES Pedro Guillén, n (%)	194 (27.7)
Weight (kg)	59.3 ± 15.0
Height (m)	1.61 ± 0.8
BMI (kg/m^2^)	22.7 ± 4.7
WC (cm)	72.9 ± 10.5
FAS-III (score)	8.1 ± 2.0
YAP-S Physical activity (score)	2.6 ± 0.7
YAP-S Sedentary behaviors (score)	2.6 ± 0.6
Sleep duration (min)	492.1 ± 55.3
Tobacco smoking, n (%)	55 (7.9)
Alcohol consumption, n (%)	124 (18.6)
Total energy intake (Kcal/day)	3028.1 ± 2019.7
MedDiet adherence	
Low n (%)	86 (12.3)
Medium n (%)	353 (50.4)
High n (%)	261 (37.3)
KIDMED (score)	6.5 ± 2.6
Psychosocial health problems (SDQ)	
Total difficulties n (%) ^a^	94 (13.4)
Total difficulties (score)	11.9 ± 6.2
Emotional symptoms n (%) ^a^	110 (15.7)
Emotional symptoms (score)	3.5 ± 2.7
Conduct problems n (%) ^a^	83 (11.9)
Conduct problems (score)	2.1 ± 1.8
Hyperactivity n (%) ^a^	104 (14.9)
Hyperactivity (score)	4.2 ± 2.2
Peer problems n (%) ^a^	43 (6.1)
Peer problems (score)	2.2 ± 1.8
Pro-social behavior n (%) ^a^	0
Pro-social behavior (score)	7.8 ± 2.1

Data are represented as the mean (standard deviation) or count (percentages). **^a^** Frequency of adolescence presenting the outcome in each category of the exposure variable were showed by percentages in parentheses. BMI: body mass index; CE: Cooperativa de Enseñanza; FAS-III: Family Affluence Scale-III; IES: Instituto de Educación Secunaria; KIDMED: Mediterranean Diet Quality Index for children and teenagers; MedDiet: Mediterranean diet; SDQ: Strengths and Difficulties Questionnaire; WC: waist circumference; YAP-S: Spanish Youth Active Profile. For the SDQ total difficulties score, the following cutoff points were applied: no psychosocial health problems (normal and borderline; range 0–17 points) and psychosocial health problems (abnormal; 17–40 points). The total score for each subscale is also provided.

**Table 2:** Association between different food groups included in the KIDMED questionnaire and psychosocial health problems (SDQ subscales) among Spanish adolescents.

	*R* ^2^	*B*	SE	*β*	*p*
Emotional problems (score)	0.142				
Has fresh or cooked vegetables more than once a day		0.418	0.218	0.073	0.056
Consumes fish regularly (at least 2–3/week)		−0.355	0.212	−0.064	0.095
Consumes nuts regularly (at least 2–3/week)		**−0.755**	**0.208**	**−0.138**	**<0.001**
Skips breakfast		**0.636**	**0.291**	**0.086**	**0.029**
Conduct problems (score)	0.111				
Takes a fruit or fruit juice every day		**−0.326**	**0.152**	**−0.084**	**0.032**
Consumes pasta or rice almost every day (≥5/week)		**0.320**	**0.135**	**0.092**	**0.019**
Skips breakfast		**0.430**	**0.190**	**0.091**	**0.024**
Hyperactivity problems (score)	0.083				
Takes sweets and candy several times every day		**0.557**	**0.215**	**0.105**	**0.010**
Has a second fruit every day		**−0.588**	**0.178**	**−0.132**	**<0.001**
Peer problems (score)	0.053				
Has fresh or cooked vegetables more than once a day		**0.406**	**0.151**	**0.108**	**0.007**
Consumes fish regularly (at least 2–3/week)		−0.251	0.146	−0.069	0.086
Skips breakfast		0.363	0.201	0.074	0.072
Pro-social scale (score)	0.090				
Likes pulses and eats them >1/week		**0.720**	**0.184**	**0.153**	**<0.001**
SDQ total difficulties (score)	0.120				
Takes a fruit or fruit juice every day		**−1.114**	**0.540**	**−0.082**	**0.039**
Has fresh or cooked vegetables more than once a day		**1.106**	**0.496**	**0.087**	**0.026**
Consumes fish regularly (at least 2–3/week)		−0.881	0.480	−0.072	0.067
Consumes nuts regularly (at least 2–3/week)		**−1.212**	**0.472**	**−0.099**	**0.010**
Skips breakfast		**1.649**	**0.661**	**0.100**	**0.013**

*B*: unstandardized beta coefficient; *β*: standardized beta coefficient; KIDMED: Mediterranean Diet Quality Index for children and teenagers; SE: standard error. Analyses were fully adjusted for age, sex, waist circumference, sleep duration, socioeconomic status, physical activity, sedentary behaviors, alcohol consumption, and tobacco consumption. The *p* values in bold indicate statistical significance for the corresponding predictor in the model (*p* < 0.05).

## Data Availability

The data used in this review are available from the corresponding authors after reasonable request.
